# Efficacy and safety of pulsed field ablation for cavotricuspid isthmus-dependent atrial flutter: A meta-analysis based on real-world evidence

**DOI:** 10.1016/j.hroo.2026.04.030

**Published:** 2026-05-13

**Authors:** Lei Liu, Aiyue Chen, Linqi Liu, Pakezhati Maimaitijiang, Xinyi Wang, Lihui Zheng

**Affiliations:** National Clinical Research Center of Cardiovascular Diseases, National Center for Cardiovascular Diseases, Fuwai Hospital, Chinese Academy of Medical Sciences and Peking Union Medical College, Beijing, People's Republic of China

**Keywords:** Pulsed field ablation, Atrial flutter, Cavotricuspid isthmus, Coronary artery spasm, Nitroglycerin, Meta-analysis

## Abstract

**Background:**

Pulsed field ablation (PFA) is effective for atrial fibrillation, but its real-world performance for cavotricuspid isthmus (CTI)-dependent atrial flutter remains inadequately defined.

**Objective:**

The purpose of this study was to systematically evaluate the efficacy and safety of PFA for CTI ablation using real-world evidence.

**Methods:**

A total of 9 observational studies (656 patients) published up to January 2026 were analyzed using a random-effects model.

**Results:**

Pooled analysis revealed a short-term bidirectional CTI block rate of 99.4% and a short-to-midterm recurrence rate of 2.14%. Mean CTI ablation time was highly efficient (8.86 minutes). Incidences of catastrophic complications and permanent atrioventricular block were 0%. The overall incidence of intraprocedural right coronary artery spasm was 2.0%. Prophylactic high-dose intravenous nitroglycerin (>1 mg) significantly reduced coronary spasm incidence compared with low-dose (≤1 mg) regimens (1% vs 8%; *P* = .0016). Sensitivity analyses confirmed spasm risk was independent of PFA catheter configuration (*P* = .9549).

**Conclusion:**

PFA is a rapid, safe, and highly effective modality for CTI ablation. The risk of transient coronary spasm may be mitigated with prophylactic high-dose nitroglycerin. These hypothesis-generating findings warrant further validation.

## Central Illustration

**Figure:**
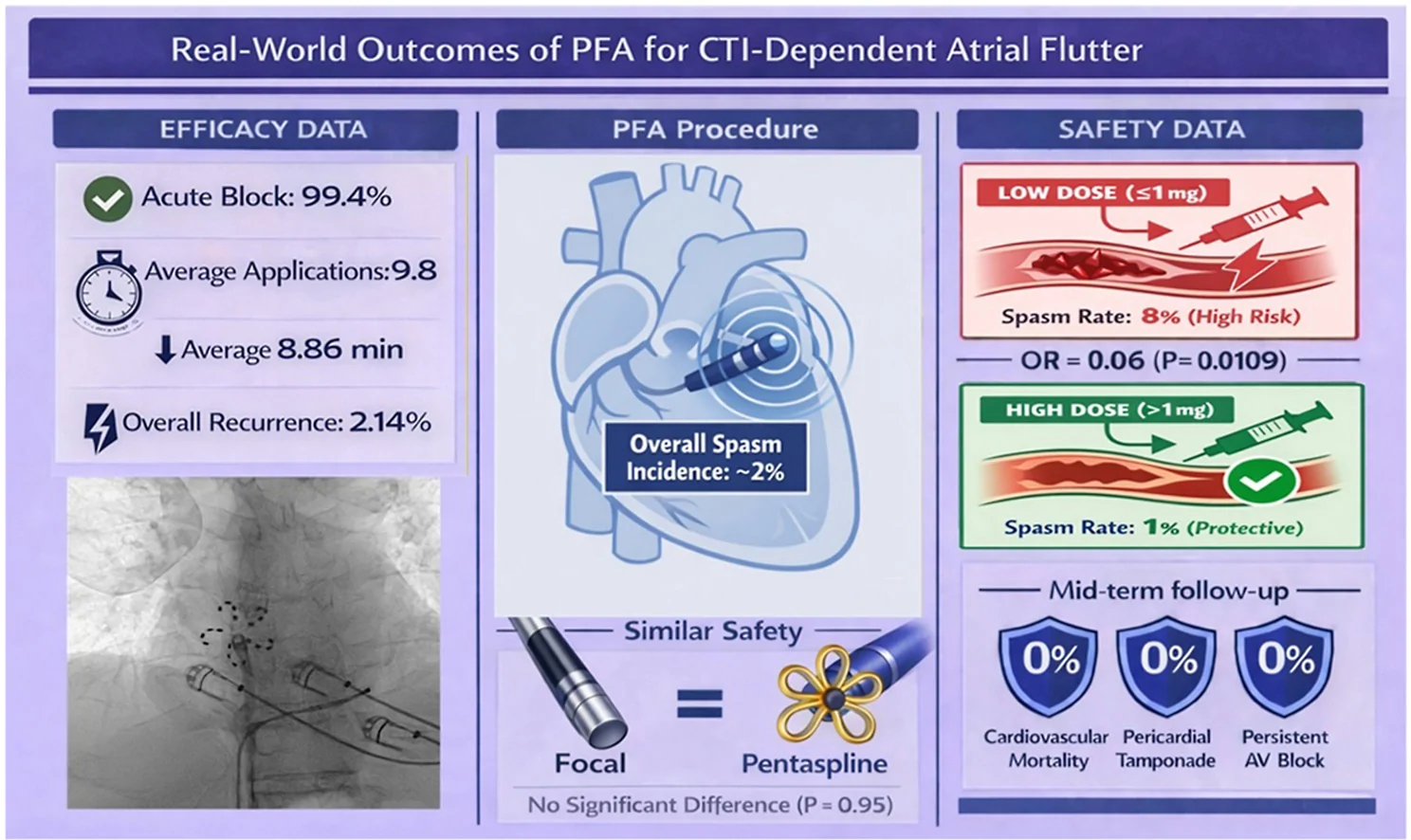

**Key Findings**

▪ Real-world evidence indicates that pulsed field ablation (PFA) is highly effective for cavotricuspid isthmus (CTI) ablation, achieving a 99.4% short-term success rate with a highly efficient average ablation time of <9 minutes.
▪ PFA exhibits an excellent safety profile for CTI ablation, with 0% pooled incidences of catastrophic complications and permanent atrioventricular block across 656 patients.
▪ The overall risk of transient intraprocedural right coronary artery spasm is low (2.0%) and can be significantly mitigated by implementing a prophylactic high-dose (>1 mg) intravenous nitroglycerin regimen.

## Introduction

Cavotricuspid isthmus (CTI)-dependent atrial flutter is a common macro‒re-entrant arrhythmia,^1^^,^^2^ often coexisting with atrial fibrillation or occurring independently. Catheter ablation aimed at achieving durable, bidirectional CTI block is recommended as the first-line therapy in international guidelines.^3^^,^^4^ Traditionally, this procedure has primarily relied on thermal energy sources, such as radiofrequency ablation (RFA) and cryoablation.^5^^,^^6^ Although generally effective, thermal modalities are limited by their nonselective mechanisms. RFA is associated with prolonged procedure times, patient discomfort, and the potential for collateral thermal damage to adjacent structures, such as the right coronary artery (RCA) and the atrioventricular node.^7^^,^^8^ In contrast, cryoablation is highly susceptible to the "heat-sink effect" from local blood flow, which can compromise lesion transmurality and continuity.^9^^,^^10^

PFA has recently emerged as a groundbreaking, nonthermal energy modality.^11^, ^12^, ^13^ By delivering microsecond-scale, high-voltage electric fields, PFA induces irreversible electroporation, offering exceptional tissue selectivity that targets myocardium while sparing vital surrounding structures such as nerves and blood vessels.^14^, ^15^, ^16^ Owing to its favorable safety profile, procedural efficiency, and promising short-to-midterm efficacy, PFA has become widely integrated into the clinical management of atrial fibrillation. Consequently, its application for CTI ablation is rapidly gaining clinical traction.^17^, ^18^, ^19^, ^20^

Despite these theoretical advantages, the precise efficacy and safety profile of PFA for CTI ablation remain insufficiently defined.^8^^,^^21^, ^22^, ^23^ Clinical evidence is primarily derived from small-scale, observational studies. Moreover, the unique biophysical properties of PFA have raised new procedural concerns. Given the close anatomical proximity of the CTI to the RCA, the high-voltage electric field can directly stimulate vascular smooth muscle, potentially triggering transient coronary artery spasm.^24^^,^^25^ The exact incidence of this phenomenon, the short-to-midterm durability of PFA-induced CTI block, and the optimal periprocedural mitigation strategies, including prophylactic nitroglycerin (NTG) regimens and catheter platform selection, are not well understood.

Therefore, this study aimed to conduct a comprehensive meta-analysis based on real-world evidence to systematically evaluate the efficacy, safety, and procedural efficiency of PFA for CTI-dependent atrial flutter. By synthesizing available observational data, we sought to assess the risk of periprocedural coronary spasm and identify key pharmacologic interventions, providing robust evidence to optimize and standardize PFA protocols in contemporary electrophysiological practice.

## Materials and methods

This study was conducted in accordance with the methodologic guidance outlined in the *Cochrane Handbook for Systematic Reviews* and was reported following the Preferred Reporting Items for Systematic Reviews and Meta-Analyses 2020 statement.^26^^,^^27^ The study protocol was prospectively registered with the National Institute for Health and Care Research (International Prospective Register of Systematic Reviews ID: CRD420251247843) and was performed without deviations from the registered protocol. Because this meta-analysis was based solely on previously published data, ethical approval and informed consent were not required.

### Search strategy

The search strategy was developed by 3 investigators (L.L., A.Y.C., and L.Q.L.). A comprehensive and independent literature search was conducted in MEDLINE (through PubMed), Web of Science, Embase, and the Cochrane Library from database inception to January 7, 2026. The search was limited to English-language publications. No restrictions were applied regarding publication date, status, or year of dissemination.

Search terms combined free-text keywords and Medical Subject Headings related to atrial flutter, cavotricuspid isthmus, and PFA. To enhance completeness, additional sources were screened, including ClinicalTrials.gov, the Epistemonikos database, and Google Scholar. Backward and forward citation tracking was performed using the *citationchaser* R package to ensure identification of all potentially eligible studies.

### Eligibility criteria

#### Inclusion criteria

Eligible studies were strictly limited to real-world observational research, such as prospective and retrospective cohort studies, clinical registries, and large case series (defined as ≥10 patients). This specific design restriction was prespecified to capture broad, unselected patient populations and reflect routine clinical practice, complementing the highly controlled environments of randomized trials. Studies were required to enroll adult patients (aged ≥18 years) with electrophysiologically confirmed CTI dependent atrial flutter, regardless of the presence of concomitant atrial fibrillation. The primary intervention had to be CTI ablation using any commercially available PFA system.

To be eligible, studies were required to report ≥1 prespecified efficacy or safety outcome. Efficacy outcomes included acute CTI block rate, first-pass block rate, short-to-midterm atrial flutter recurrence, number of PFA applications, CTI ablation time, and need for adjunctive RFA. Safety outcomes included intraprocedural coronary artery spasm, atrioventricular block, pericardial effusion or cardiac tamponade, all-cause mortality, use of prophylactic NTG, and total procedure time.

#### Exclusion criteria

The following records were excluded: (1) non–real-world studies, including animal or in vitro experiments, and randomized controlled trials (RCTs) in which PFA-specific data could not be extracted separately; (2) studies evaluating non–CTI-dependent atrial flutter; (3) studies using non-PFA modalities (eg, exclusive radiofrequency or cryoablation) for CTI ablation; (4) studies lacking extractable prespecified efficacy or safety outcomes; (5) case reports or small case series (<10 patients), to reduce small-study bias; (6) reviews, editorials, commentaries, and conference abstracts; (7) non-English publications; and (8) duplicate or overlapping reports.

### Study end points

To systematically evaluate the clinical performance of PFA for CTI-dependent atrial flutter, predefined efficacy, safety, and procedural end points were established as follows.

#### Efficacy end points

Acute CTI block rate was defined as the proportion of patients in whom bidirectional conduction block across the CTI was achieved at the end of the ablation procedure. First-pass success rate was defined as the proportion of patients in whom bidirectional CTI block was achieved after a single initial sequence of PFA applications without requiring additional touch-up ablation. Short-to-midterm atrial flutter recurrence rate was defined as the proportion of patients with documented recurrence of atrial flutter or related atrial tachyarrhythmias during follow-up, confirmed by electrocardiography or Holter monitoring.

#### Safety end points

Intraprocedural coronary artery spasm, the primary safety outcome of this meta-analysis, was defined as RCA spasm occurring during or immediately after PFA energy delivery, confirmed by coronary angiography or accompanied by characteristic ischemic ECG changes (eg, ST-segment elevation). Other procedural complications included new-onset atrioventricular (AV) block attributable to ablation, pericardial effusion or cardiac tamponade requiring intervention, and all-cause or cardiovascular mortality.

#### Procedural metrics

Number of PFA applications was defined as the total number of discrete energy deliveries required to achieve and consolidate bidirectional CTI block in an individual patient. CTI ablation time was defined as the duration (in minutes) from the first PFA energy delivery to confirmation of complete bidirectional CTI block.

### Study selection

All records identified through the comprehensive literature search were imported into a reference management system, and initial electronic duplicates were removed. To rigorously exclude the double-counting of patient populations, we systematically cross-evaluated author affiliations, study recruitment periods, and center names during the full-text screening stage, explicitly excluding any overlapping cohorts. Study selection was performed in 2 stages. First, 2 investigators independently screened titles and abstracts to identify potentially eligible studies. Second, full-text articles of relevant records were retrieved and assessed against the predefined inclusion and exclusion criteria. Disagreements between reviewers were resolved through discussion and consensus. If consensus could not be achieved, a third senior investigator made the final decision. When outcome data was incomplete or unclear, corresponding authors were contacted to obtain additional information. The selection process was fully documented, including explicit reasons for exclusion at the full-text stage. Borderline inclusion decisions were further evaluated in sensitivity analyses to ensure the methodologic robustness of the final study cohort.

### Data extraction

Data extraction was conducted independently by 2 investigators (L.L. and A.Y.C.) using a prespecified, standardized electronic data collection form. Discrepancies were resolved through crosschecking and discussion to reach consensus; unresolved disagreements were adjudicated by a third senior investigator (L.Q.L.).

To support quantitative synthesis and planned subgroup analyses, data were extracted across the following domains: (1) study characteristics (first author, publication year, study design, sample size, and mean follow-up duration); (2) baseline patient characteristics (demographics and key comorbidities); (3) procedural and technical parameters (catheter platform [eg, focal vs pentaspline], prophylactic NTG use and dose category [≤1 mg vs >1 mg], number of PFA applications, and CTI ablation time); (4) efficacy outcomes (event counts for acute bidirectional CTI block, first-pass success, and atrial flutter recurrence during follow-up); and (5) safety outcomes (intraprocedural coronary artery spasm events and other major periprocedural complications, including new-onset AV block, pericardial effusion or cardiac tamponade, and mortality).

### Quality assessment

The methodologic quality and risk of bias of the included observational studies were independently assessed by 2 investigators. Given the real-world design of the included evidence, the 10-domain Joanna Briggs Institute Critical Appraisal Checklist was applied to each study. Discrepancies in quality assessment were resolved through discussion, and a third senior investigator was consulted when necessary to achieve consensus.

To enhance transparency, a traffic light plot was generated to visually summarize the quality judgments across all 10 Joanna Briggs Institute domains. These domains evaluate key methodologic aspects, including clarity of inclusion criteria, reliability of condition measurement, and appropriateness of statistical analyses. This structured appraisal established the methodologic foundation of the included studies and informed the interpretation of findings in subsequent sensitivity analyses.

### Statistical analysis

All quantitative syntheses were conducted using R statistical software (R version 4.5.1; R Foundation for Statistical Computing, Vienna, Austria) with the *meta* package. Given the expected clinical and methodologic heterogeneity inherent to real-world observational studies, random-effects models were applied throughout to generate conservative pooled estimates. For dichotomous outcomes, pooled proportions with 95% confidence intervals (CIs) were calculated. To properly handle zero-event studies (eg, concerning pericardial effusion or AV block) and stabilize variances when event rates approached 0% or 100%, the Freeman-Tukey double arcsine transformation was applied before data pooling to reduce extreme-value distortion. In parallel, pooled estimates were also calculated using a generalized linear mixed model to enhance analytical robustness. For comparative outcomes, pooled odds ratios with 95% CIs were computed. For continuous outcomes, means and standard deviations were extracted to calculate pooled mean values with corresponding 95% CIs. Between-study heterogeneity was assessed using Cochran’s Q test (*P* < .10 indicating statistical significance) and the I^2^ statistic, with I^2^ >50% considered substantial heterogeneity. Subgroup analyses were conducted to explore potential sources of heterogeneity, focusing on clinically relevant factors such as catheter type. Moreover, a data-driven subgroup analysis stratified by NTG dosage (≤1 mg vs >1 mg) was performed; this specific 1-mg threshold was not prespecified on the basis of a uniform pharmacologic rationale but rather determined by the distinct administration protocols reported across the included centers. Differences among subgroups were evaluated using the Q test for subgroup interaction. To further assess the stability of pooled estimates and identify influential studies, leave-one-out sensitivity analyses were performed. Of note, formal assessments of publication bias (eg, funnel plots or Egger's test) were not conducted, given the total number of included studies (N = 9) was below the Cochrane-recommended threshold of 10. All statistical tests were 2-sided, and *P* < .05 was considered statistically significant.

## Results

### Study selection and characteristics

The systematic literature search identified 324 records from the selected electronic databases: PubMed or MEDLINE (107), Embase (107), Web of Science (81), and the Cochrane Library (29). After removing 145 duplicates, 179 unique articles underwent title and abstract screening. During this stage, 104 records were excluded, including 93 that did not meet the inclusion criteria and 11 that were not real-world studies. Subsequently, 68 full-text articles were assessed for eligibility. Of these, 59 were excluded for the following reasons: lack of energy source–specific data (n = 22), insufficient outcome data (n = 12), absence of PFA application for CTI-dependent atrial flutter (n = 10), overlapping cohorts (n = 8), and concerns regarding study quality (n = 7). Ultimately, 9 studies fulfilled all eligibility criteria and were included in the final quantitative meta-analysis. The study selection process is detailed in the Preferred Reporting Items for Systematic Reviews and Meta-Analyses flow diagram (Supplemental Figure 1).

The baseline characteristics of the included studies are listed in Table 1. All studies were conducted in real-world clinical settings and included both prospective and retrospective designs published between 2022 and 2026. A total of 656 patients who underwent PFA for CTI-dependent atrial flutter were analyzed. The study populations were predominantly male, with proportions ranging from 63.2%‒84.4% among studies reporting sex distribution. The mean or median age ranged from 62.0‒69.5 years. Regarding the PFA technology used, pentaspline catheters were the most used (5 studies), followed by focal PFA catheters (3 studies) and the varipulse catheter (1 study). The need for adjunctive radiofrequency touch-up ablation to achieve complete CTI block was low across studies (0.0%–7.6%), reflecting the high primary procedural efficacy of PFA. Follow-up duration ranged from 90–65 days.

**Table 1:** Baseline characteristics of the included studies

Study ID	Region	Study Setting	Study Design	Sample Size (n)	Age, y∗	Male, n (%)	CHA_2_DS_2_-VASc Score	Ablation Catheter	Touch-up Ablation,† n (%)	Follow-up (d)
Farnir et al (2025)^8^	The Netherlands, Denmark	Real-world study	Prospective	82	66.0 ± 8.0	67 (81.7%)	1.6	Focal	2 (2.4%)	90
Demian et al (2025)^22^	United States	Real-world study	Prospective	207	NR	NR	NR	Pentaspline	1 (0.5%)	181.5
Chaumont et al. (2024)^21^	France	Real-world study	Prospective	32	62.0 ± 8.0	27 (84.4%)	2	Pentaspline	0 (0.0%)	NR
Rodriguez-Riascos et al (2024)^23^	United States	Real-world study	Retrospective	132	69.5 (64-76)	96 (72.7%)	3	Pentaspline	10 (7.6%)	120.1
Tam et al (2025)^25^	Hong Kong, China	Real-world study	Prospective	19	62.9 ± 10.1	12 (63.2%)	2	Pentaspline	0 (0.0%)	90
Reddy et al (2022)^28^	Czech Republic	Real-world study	Prospective	20	65.1 ± 7.7	14 (70.0%)	2.4	Pentaspline	0 (0.0%)	NR
Andria et al (2026)^29^	Israel	Real-world study	Retrospective	11	NR	NR	NR	Varipulse	0 (0.0%)	NR
Ruwald et al (2024)^30^	Denmark	Real-world study	Prospective	12	NR	NR	NR	Focal	0 (0.0%)	NR
Reddy et al (2025)^31^	Global (multicenter)	Real-world study	Prospective	141	NR	NR	NR	Focal	0 (0.0%)	365

Data are presented as mean ± standard deviation, median (interquartile range), or number (percentage).

CHA_2_DS_2_-VASc = congestive heart failure, hypertension, age ≥75 (doubled), diabetes, stroke (doubled), vascular disease, age 65 to 74 and sex category (female); NR = not reported.

∗ Age is presented as mean ± standard deviation or median (interquartile range) depending on the original study report.

† Touch-up ablation refers to additional radiofrequency ablation applications required to achieve complete cavotricuspid isthmus block after pulsed field ablation.

The risk of bias assessments for the included studies are presented in Supplemental Figure 2. Overall, the methodologic quality of the included evidence was high. Only 1 study was judged to have some concerns, specifically related to consecutive participant inclusion.

### Efficacy outcomes

#### Acute CTI block and first-pass success

**Figure 1:**
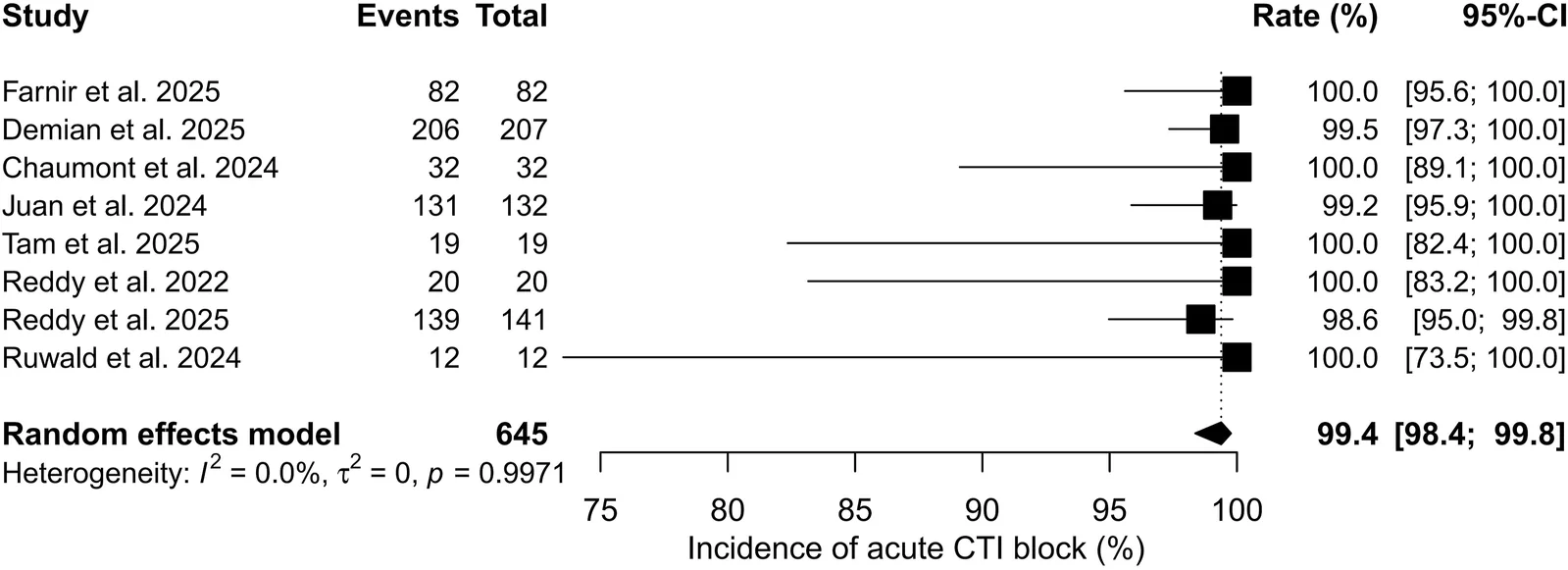
Forest plot of the acute CTI block rate. Pooled incidence of acute CTI block after PFA across all included studies. *Black squares* represent the event rate of individual studies, with the size of the square proportional to the study's statistical weight. *Horizontal lines* indicate 95% CIs. The *black diamond* represents the pooled event rate and 95% CI based on the random-effects model. CI = confidence interval; CTI = cavotricuspid isthmus.

Overall, 8 studies, including a total of 645 patients, reported the incidence of acute bidirectional CTI block after PFA. In the random-effects meta-analysis, PFA achieved a pooled acute CTI block rate of 99.4% (95% CI: 98.4%–99.8%). No statistical heterogeneity was observed (I^2^ = 0.0%, *P* = .9971), indicating highly consistent procedural efficacy across centers and operators (Figure 1).

Only 3 studies reported first-pass CTI block rates. Given the limited number of studies and substantial variability in sample sizes and procedural protocols, a quantitative synthesis was not performed. Instead, a narrative summary is provided. First-pass success ranged from 89%–100%. In the largest cohort, Farnir et al^8^ (N = 82) reported a first-pass success rate of 93%. Chaumont et al^21^ (N = 32) observed a rate of 89%. Ruwald et al^30^ (N = 12) reported a 100% first-pass success; however, this estimate should be interpreted cautiously owing to the small sample size. Overall, despite interstudy variability, the available evidence consistently suggests a high likelihood of achieving acute bidirectional CTI block with PFA on the first attempt.

#### Short-to-midterm atrial flutter recurrence

**Figure 2:**
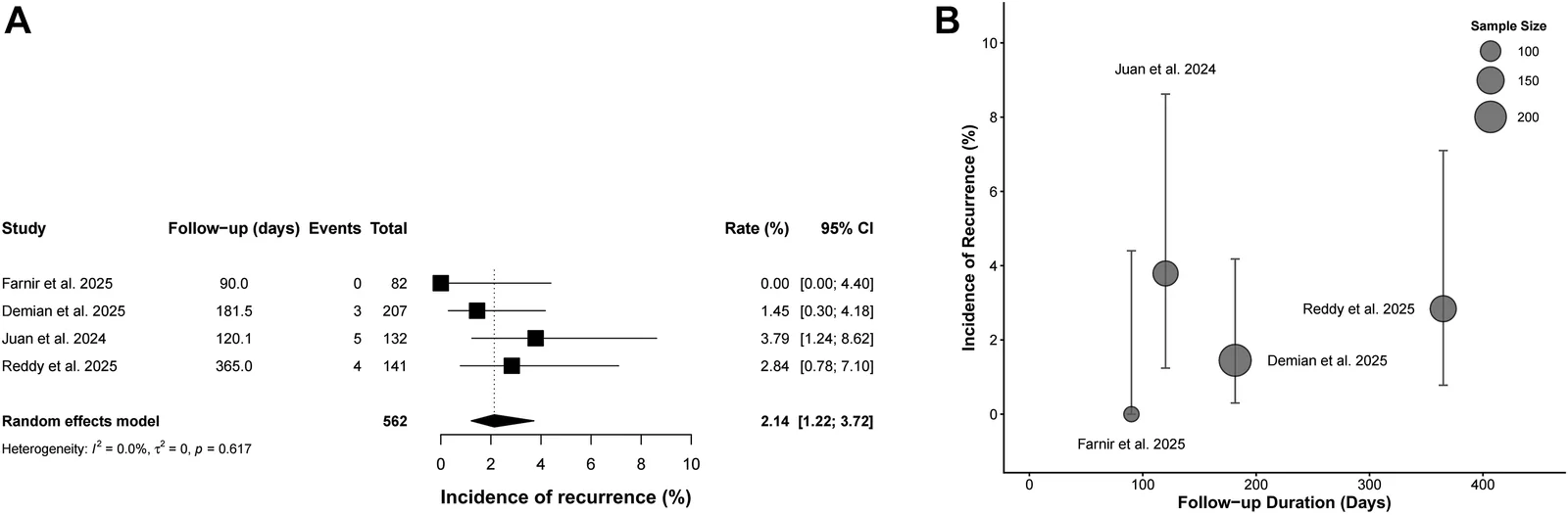
Incidence of atrial flutter recurrence after pulsed field ablation and its relationship with follow-up duration. **A:** Forest plot detailing the pooled incidence of recurrence across the included studies. **B:** Bubble plot illustrating the relationship between mean follow-up duration (days) and the incidence of recurrence (%). The *bubble* area is proportional to the study's sample size, and the *vertical lines* represent the 95% CIs for each study. CI = confidence interval.

Short-to-midterm freedom from atrial flutter recurrence is a key indicator of procedural durability. 4 studies, including 562 patients, reported arrhythmia recurrence during follow-up. The pooled recurrence rate was 2.14% (95% CI: 1.22%–3.72%), indicating a low incidence of postprocedural atrial flutter. No significant heterogeneity was observed (I^2^ = 0.0%, *P* = 0.617), suggesting consistent short-to-midterm efficacy of PFA across different cohorts (Figure 2A).

We descriptively mapped recurrence incidence against mean follow-up time (90–365 days) using a bubble plot. Recurrence ranged from 0.00% at 90 days (Farnir et al^8^) to 2.84% at 1 year.^31^ Although this visualization illustrates persistently low recurrence without an obvious temporal trend, it must be emphasized that with only 4 contributing data points, this plot (Figure 2B) has limited inferential value and precludes robust conclusions regarding the impact of follow-up duration. These findings suggest that the acute CTI block achieved with PFA translates into sustained midterm rhythm control.

### Safety outcomes

#### Intraprocedural coronary artery spasm

**Figure 3:**
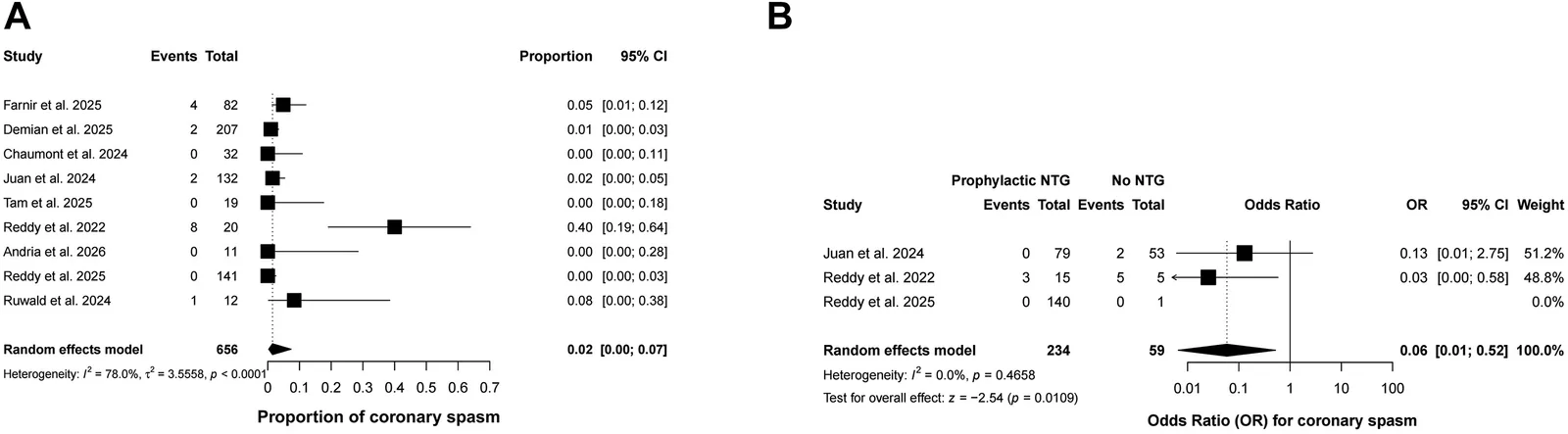
Incidence of intraprocedural coronary artery spasm and the effect of prophylactic nitroglycerin. **A:** Forest plot showing the pooled incidence of coronary artery spasm during pulsed field ablation across all included studies. The overall incidence was 2% (95% CI: 0.00–0.07). Significant heterogeneity was observed (I^2^ = 78.0%, *P* < .0001), largely driven by the study by Reddy et al.^28^**B:** Forest plot comparing the risk of coronary artery spasm in patients who received prophylactic (NTG) and those who did not. A random-effects model using the Mantel–Haenszel method was applied. Prophylactic NTG was associated with a significant reduction in spasm risk (OR: 0.06; 95% CI: 0.01–0.52; *P* = .0109). No heterogeneity was detected in this comparison (I^2^ = 0.0%, *P* = .466). CI = confidence interval; NTG = nitroglycerin; OR = odds ratio.

In 9 studies including 656 patients, intraprocedural coronary artery spasm was reported during PFA for CTI-dependent atrial flutter. In the random-effects meta-analysis, the pooled incidence of coronary spasm was 2% (95% CI: 0%–7%). However, substantial heterogeneity was observed (I^2^ = 78.0%, *P* < .001), with reported incidences ranging from 0%–7%.^28^ Notably, a sensitivity analysis excluding this outlier was performed. As detailed in the supplementary material (Supplemental Figure 3B), excluding the Reddy et al^28^ cohort refined the pooled incidence of coronary spasm to 1% (95% CI: 0%–3%) and dramatically reduced statistical heterogeneity (I^2^ = 0%). This marked variability highlights the importance of identifying procedural or pharmacologic strategies to mitigate this complication (Figure 3A).

To evaluate the preventive effect of pharmacologic prophylaxis, we compared patients who received prophylactic NTG with those who did not; 3 studies involving 293 patients (234 in the NTG group and 59 in the control group) were included in this analysis. Prophylactic NTG administration was associated with a significant reduction in the risk of coronary spasm, with a pooled odds ratio of 0.06 (95% CI: 0.01–0.52; *P* = .0109), corresponding to a 94% relative risk reduction. No heterogeneity was detected (I^2^ = 0.0%, *P* = .466), indicating a consistent protective effect of NTG during PFA of the cavotricuspid isthmus (Figure 3B).

#### Subgroup analyses: NTG dosage and catheter type

**Figure 4:**
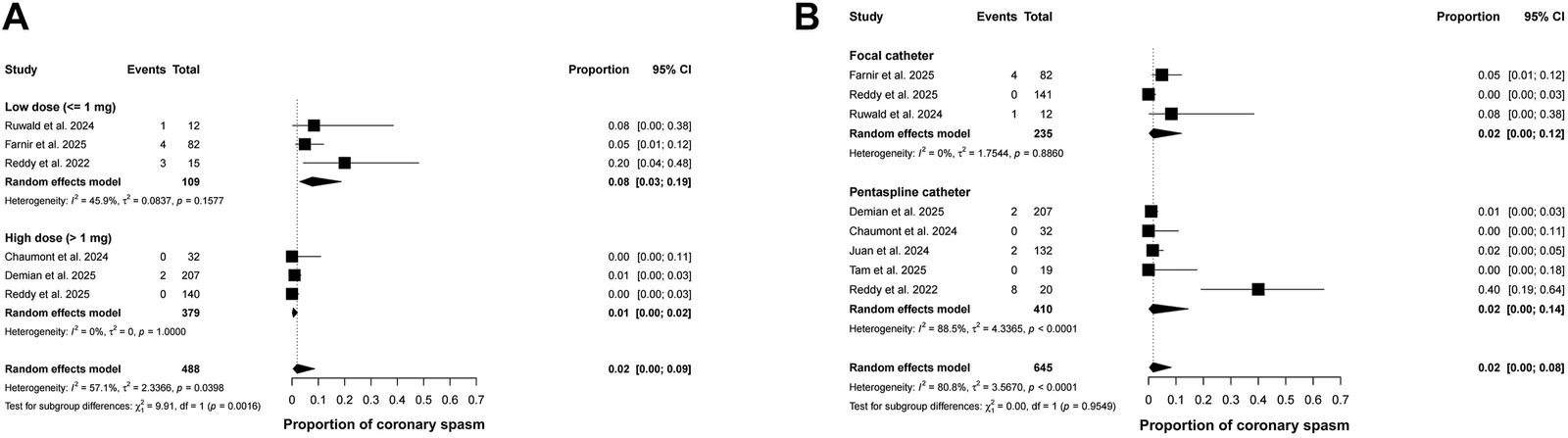
Subgroup analyses of intraprocedural coronary artery spasm according to nitroglycerin dosage and catheter type. **A:** Forest plot showing the pooled incidence of coronary artery spasm stratified by prophylactic nitroglycerin dose (low dose: ≤1 mg vs high dose: >1 mg). A significant subgroup difference was observed (*P* = .0016), indicating a lower spasm incidence with the high-dose strategy. **B:** Forest plot illustrating the pooled incidence of coronary artery spasm according to pulsed field ablation catheter type (focal vs pentaspline). No significant difference was detected between catheter designs (*P* = .9549). CI = confidence interval.

To identify the optimal preventive strategy for intraprocedural coronary artery spasm, a subgroup analysis stratified by prophylactic NTG dosage was conducted. The pooled incidence of coronary spasm was significantly lower in patients receiving a high NTG dose (>1 mg) than in those receiving a low dose (≤1 mg) (1% [95% CI: 0.00–0.02] vs 8% [95% CI: 0.03–0.19]; P for subgroup difference = .0016) (Figure 4A). Importantly, the substantial heterogeneity observed in the overall analysis was eliminated within the high-dose subgroup (I^2^ = 0%, *P* = 1.000), suggesting that inadequate NTG dosing was a major contributor to the observed variability.

We further evaluated whether catheter design influenced the risk of coronary spasm. Subgroup analysis showed no significant difference between procedures performed with focal catheters and those using pentaspline catheters (2% [95% CI: 0.00–0.12] vs 2% [95% CI: 0.00–0.14]; P for subgroup difference = .9549) (Figure 4B). These findings indicate that coronary spasm appears to be independent of catheter type and is more closely related to pharmacologic prophylaxis.

#### Sensitivity analyses: Robustness of coronary spasm findings

To evaluate the robustness of the findings related to intraprocedural coronary artery spasm, a series of sensitivity analyses was performed.

First, a leave-one-out analysis was conducted to assess the stability of the pooled incidence estimate (Supplemental Table 1). Sequential exclusion of individual studies did not materially change the overall incidence, confirming the robustness of the primary outcome. Notably, removal of the study by Reddy et al^28^ substantially reduced heterogeneity (I^2^ decreased from 78.0% to 34.4%), identifying this study as the primary contributor to between-study variability.

Additional sensitivity analyses were performed to validate the subgroup findings (Supplemental Figure 3). When evaluating prophylactic NTG dosage, the results remained consistent after modifying inclusion criteria. The pooled incidence of coronary spasm continued to be significantly lower in the high-dose group (>1 mg) than in the low-dose group (≤1 mg) (1% vs 5%; P for subgroup difference = .0095) (Supplemental Figure 3A).

A further sensitivity analysis evaluated catheter type by excluding the previously mentioned outlier study^28^ and incorporating a third catheter category (varipulse). After exclusion, heterogeneity was eliminated (I^2^ = 0%, *P* = .591). Consistent with the primary subgroup analysis, no significant differences in spasm incidence were observed among focal, pentaspline, and varipulse catheter systems (P for subgroup difference = .8807) (Supplemental Figure 3B).

Collectively, these analyses confirm the statistical stability of the overall estimates and indicate that adequate NTG dosing, rather than catheter platform, is the principal factor associated with reduced coronary artery spasm during PFA.

#### Other periprocedural complications

In addition to coronary artery spasm, other major procedural complications were evaluated to comprehensively characterize the safety profile of PFA for CTI-dependent atrial flutter (Supplemental Figure 4).

9 studies including 644 patients reported AV block events. The pooled incidence was 0% (95% CI: 0.00–0.01), with minimal heterogeneity (I^2^ = 6.0%, *P* = .384) (Supplemental Figure 4A). Only 2 studies (Farnir et al^8^; Rodriguez-Riascos et al^23^) documented isolated AV block events (2 cases each). This very low incidence suggests a favorable safety margin of PFA when energy delivery occurs at CTI.

Severe complications commonly associated with thermal ablation were not observed. Across 8 reporting studies (N = 515), there were no cases of pericardial effusion or tamponade (Supplemental Figure 4B) and no cardiovascular deaths (Supplemental Figure 4C). The pooled incidence for both outcomes was 0% (95% CI: 0.00–0.00), with no heterogeneity (I^2^ = 0.0%, *P* = .970).

Overall, these findings indicate an excellent safety profile of PFA for CTI ablation, with a negligible risk of major adverse events.

### Procedural times and ablation efficiency

To assess the intraprocedural efficiency of PFA for CTI-dependent atrial flutter, we analyzed both the number of PFA applications and total CTI ablation time.

**Figure 5:**
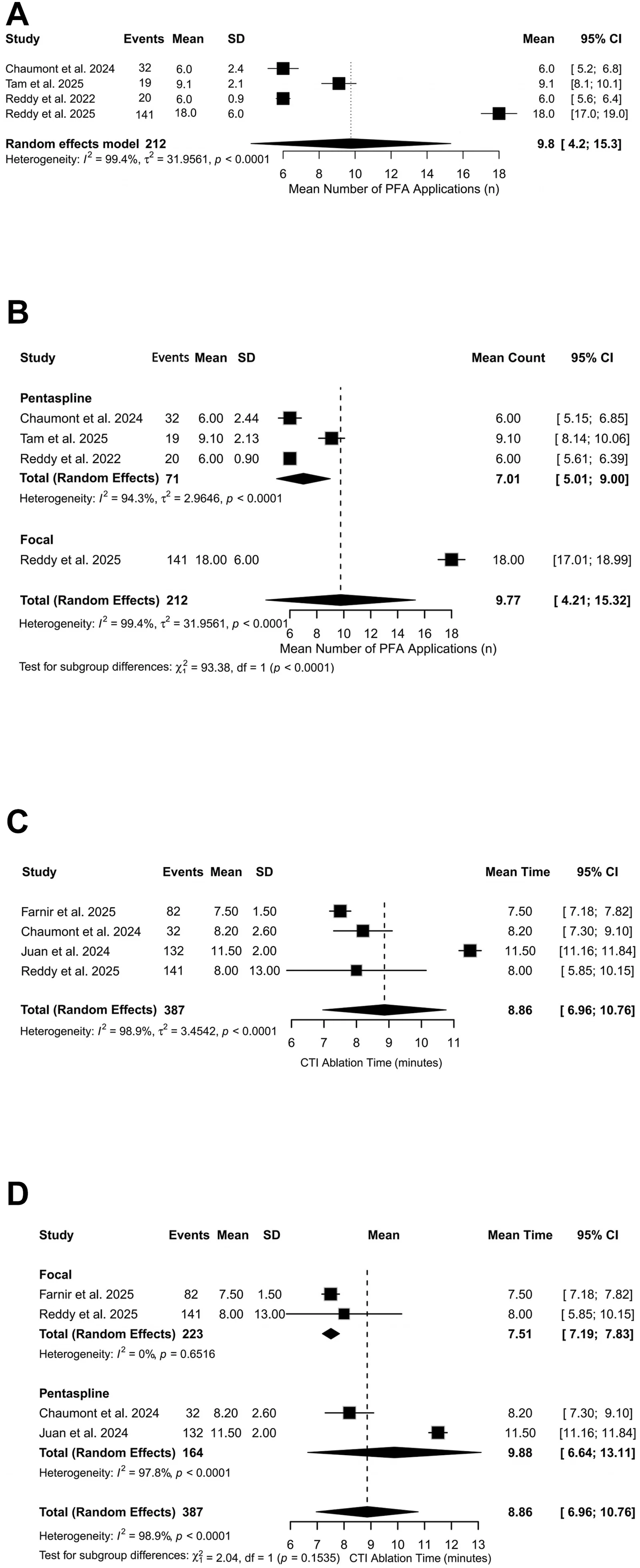
Forest plots and subgroup analyses of pulsed field ablation (PFA) applications and cavotricuspid isthmus (CTI) ablation time. **A:** Forest plot showing the pooled mean number of PFA applications across all included studies. **B:** Subgroup analysis of the mean number of PFA applications stratified by catheter type (pentaspline vs focal). **C:** Forest plot displaying the pooled mean CTI ablation time (minutes) across studies. **D:** Subgroup analysis of mean CTI ablation time according to catheter type (pentaspline vs focal). CI = confidence interval.

Data on the number of PFA applications were available from 4 studies including 212 patients. The pooled mean number of applications was 9.8 (95% CI: 4.2–15.3). As anticipated with evolving technologies, substantial heterogeneity was observed (I^2^ = 99.4%, *P* < .0001) (Figure 5A). To investigate potential sources of variability, a subgroup analysis stratified by catheter type was performed. Procedures using pentaspline catheters required significantly fewer applications (mean: 7.01 [95% CI: 5.01–9.00]) than did focal catheters (mean: 18.00 [95% CI: 17.01–18.99]), with a highly significant subgroup difference (*P* < .0001) (Figure 5B). These findings indicate that application burden is strongly influenced by catheter platform and its associated energy delivery strategy.

4 studies comprising 387 patients reported CTI ablation time. The pooled mean ablation time was 8.86 minutes (95% CI: 6.96–10.76), again with substantial heterogeneity (I^2^ = 98.9%, *P* < .0001) (Figure 5C). Subgroup analysis by catheter type showed that focal catheters achieved a mean CTI ablation time of 7.51 minutes (95% CI: 7.19–7.83). Notably, heterogeneity was completely resolved within this subgroup (I^2^ = 0%, *P* = .652). In contrast, pentaspline catheters were associated with a longer mean ablation time of 9.88 minutes (95% CI: 6.64–13.11) (Figure 5D).

Overall, these results indicate the rapid procedural performance of PFA for CTI ablation. However, both application counts and ablation time are highly dependent on the specific catheter technology used.

## Discussion

This real-world evidence-based meta-analysis systematically evaluated the clinical performance of PFA for CTI-dependent atrial flutter. First, PFA indicated exceptional clinical efficacy, achieving a 99.4% short-term bidirectional CTI block rate with an impressively low short-to-midterm arrhythmia recurrence rate of 2.14%. Second, regarding the most scrutinized complication specific to CTI PFA, the overall incidence of intraprocedural RCA spasm was maintained at a low 2%, with a complete absence of catastrophic complications such as pericardial effusion, tamponade, or mortality. Third, the prophylactic intravenous administration of high-dose (>1 mg) NTG profoundly altered the safety profile, yielding a 94% relative risk reduction for coronary spasm, a protective effect that proved consistent across different catheter platforms. Finally, although PFA exhibited high overall procedural efficiency (mean CTI ablation time of 8.86 minutes), the specific energy delivery dynamics, namely, the required number of PFA applications and exact ablation times, were fundamentally dictated by the specific catheter design (focal vs pentaspline). Collectively, these findings strongly support the high feasibility and safety of PFA as a first-line modality for CTI-dependent atrial flutter in real-world clinical practice.

The pooled analysis showed a 99.4% rate of acute bidirectional CTI block and a low atrial flutter recurrence rate of 2.14% during follow-up. These findings underscore the high efficacy of PFA in CTI ablation. Historically, CTI ablation has relied primarily on RFA and cryoablation, with RFA indicating robust historical benchmarks of >95% short-term success rates.^19^^,^^32^ However, although RFA enables controlled point-by-point lesion formation, it carries inherent risks of collateral thermal injury to adjacent structures, including the coronary arteries.^33^ Nevertheless, it remains susceptible to the heat-sink effect potentially causing incomplete or nontransmural lesions and reduced long-term durability.^34^^,^^35^ In anatomically complex CTI substrates, such as prominent trabeculations, deep pouches, or a large Eustachian ridge, thermal energy sources may fail to create continuous transmural lesions, increasing the risk of procedural failure or late recurrence.^33^^,^^36^ Furthermore, PFA, using tissue-selective nonthermal irreversible electroporation, provides an alternative energy modality that also achieves highly consistent short-term success without reliance on thermal conduction.^37^

Regarding durability, the pooled recurrence rate of 2.14% supports sustained lesion stability after PFA. This observation is consistent with early clinical experiences. For example, in patients with recurrent typical atrial flutter after multiple failed RFA procedures, PFA maintained arrhythmia-free status at 1 year without antiarrhythmic therapy.^38^ Similarly, in a cardiovascular magnetic resonance-guided CTI ablation study, the recurrence-free rate reached 82% at a median follow-up of 27 months.^39^ It should be acknowledged that long-term recurrence after CTI ablation depends not only on the energy modality but also on patient-specific factors, including structural heart disease, prior cardiac surgery, and atrial substrate characteristics.^40^^,^^41^ In selected high-risk populations, such as patients with complex congenital heart disease after atrial switch procedures, recurrence rates may approach 50%.^40^ Nonetheless, within a broad real-world atrial flutter population, the low recurrence rate observed in this meta-analysis supports PFA as an effective and durable ablation strategy.

Contextualizing our real-world findings with recent high-quality randomized data is essential. The landmark A Prospective Single Arm Open Label Study of the FARAPULSE Pulsed Field Ablation System in Subjects With Persistent Atrial Fibrillation (ADVANTAGE AF; NCT05443594) trial substudy, published after our prespecified search cutoff (January 7, 2026), indicated a highly favorable safety and efficacy profile for PFA in CTI ablation. Encouragingly, our pooled real-world short-term success rate (99.4%) and short-to-midterm recurrence rates closely mirror these rigorous trial outcomes. However, observational cohorts typically encompass broader, unselected patient populations and heterogeneous institutional protocols (eg, varying NTG administration regimens). Consequently, real-world data may yield slightly higher or more variable pooled estimates for specific periprocedural events, such as transient coronary spasm, than strictly controlled trials

Regarding overall safety, this study confirms that PFA has an excellent safety profile for the treatment of CTI-dependent atrial flutter. Traditionally, CTI ablation has relied on RFA and cryoablation. Although RFA allows controlled, point-by-point linear lesions, thermal spread can damage adjacent structures, such as the coronary arteries, phrenic nerve, or esophagus, and may cause irreversible coagulative necrosis.^33^^,^^42^ Cryoablation, although showing preliminary safety,^43^ is limited by the heat-sink effect, which can reduce lesion depth and compromise efficacy.^34^ In addition, PFA’s nonthermal, tissue-selective mechanism fundamentally avoids the direct collateral thermal injury pathways associated with traditional heating or cooling modalities.^44^ In this study, pooled incidences of pericardial effusion, cardiac tamponade, and cardiovascular mortality were zero. Even when energy is delivered near the cardiac conduction system, AV block remains rare, further supporting PFA’s favorable safety profile.

Nevertheless, the unique electric field delivery of PFA raises concerns regarding adjacent critical structures, particularly the RCA.^45^ In a prior study using a focal unipolar PFA catheter (N = 82), 5% of patients exhibited transient ST-segment elevation suggestive of RCA spasm, and 2% experienced transient complete AV block.^8^ In the present pooled analysis, the overall incidence of coronary spasm remained low at 2%, highlighting the importance of understanding its underlying mechanisms to optimize clinical practice. It must be acknowledged, however, that the observed heterogeneity in spasm incidence (I^2^ = 78.0%) is not solely attributable to variations in prophylactic NTG dosing. Differences in the ways aggressively coronary spasm was investigated across studies undoubtedly contributed to this variability. Some centers used routine coronary angiography or continuous intracardiac echocardiography monitoring, enabling the detection of asymptomatic or subclinical vasospasm. Conversely, other studies relied primarily on the detection of clinically overt events driven by pronounced ischemic electrocardiographic changes. Consequently, the pooled estimate reflects these diverse diagnostic thresholds alongside true pharmacologic effects.

Mechanistically, PFA-induced vasoconstriction results from direct electrical stimulation of vascular smooth muscle cells rather than thermal injury. High-voltage electric fields depolarize cell membranes and activate voltage-gated calcium channels, triggering rapid calcium influx.^46^ Although specific waveform adjustments can mitigate involuntary contractions,^47^^,^^48^ direct electric field effects cannot be completely eliminated. Furthermore, electroporation disrupts overall calcium homeostasis by forming nonselective membrane pores. This sharply increases cytosolic calcium, culminating in sustained smooth muscle contraction.^49^

A key finding of this study is that prophylactic NTG dose is the primary determinant in mitigating coronary spasm. It is important to note that the 1-mg NTG threshold used in our analysis was data-driven, reflecting the distinct institutional protocols, with inherent heterogeneity in NTG timing, route, and cumulative dosing, across the included studies. Nevertheless, subgroup and sensitivity analyses indicate that intravenous high-dose NTG (>1 mg) significantly reduces spasm incidence from 5% in the low-dose group (≤1 mg) to 1% (*P* = .0095). Mechanistically, NTG acts as a nitric oxide donor, activating guanylate cyclase to induce smooth muscle relaxation and counteracting the PFA-induced calcium influx and contraction.^50^ Sensitivity analyses controlling for catheter design confirmed no significant difference in spasm incidence among focal, pentaspline, or varipulse systems (*P* = .8807). This indicates that coronary spasm is primarily driven by the anatomical proximity of the CTI to the RCA and the intrinsic properties of the electric field, rather than by specific catheter designs.^8^ Taken together, these hypothesis-generating findings suggest that an intravenous bolus of high-dose (>1 mg) NTG represents a promising prophylactic strategy to minimize coronary spasm risk. However, given the observational nature and limited sample sizes of these subgroup analyses, further validation in large-scale, prospective randomized trials is warranted before its routine incorporation into standard operating procedures.

Beyond its strong safety and efficacy profile, this meta-analysis highlights the exceptional procedural efficiency of PFA for CTI ablation. The pooled mean CTI ablation time was 8.86 minutes, highlighting the rapid procedural capability of this nonthermal modality. In conventional RFA, achieving contiguous and transmural lesions across the isthmus requires prolonged energy application owing to point-by-point conductive heating and the need for catheter stability.^51^ In contrast, PFA delivers a nonthermal, near-instantaneous high-voltage electric field, accelerating this critical step.^16^ Subgroup analyses revealed that procedural efficiency is closely linked to catheter type. Procedures with pentaspline catheters required fewer PFA applications (mean: 7.01) than those with focal catheters (mean: 18.00). This difference reflects anatomical and mechanical factors: the multispline “flower” or “basket” design provides a broader ablation footprint, covering the full CTI width with fewer overlapping applications. Focal catheters, by contrast, rely on a point-by-point approach, necessitating more applications to ensure lesion continuity. Interestingly, fewer applications did not always translate into shorter ablation times. Focal catheters achieved a mean CTI ablation time of 7.51 minutes, shorter than the 9.88 minutes for pentaspline catheters. This apparent paradox likely reflects greater maneuverability and operator familiarity with single-tip focal catheters in the right atrium. Deploying a pentaspline catheter to achieve optimal multielectrode contact across the highly trabeculated CTI can require careful positioning, adding time before energy delivery. Despite these differences, both catheter platforms maintain rapid procedural performance, underscoring PFA as a highly efficient therapeutic approach.

Several limitations of this meta-analysis warrant consideration. First, our prespecified protocol restricted inclusion to single-arm observational studies to evaluate routine clinical practice, which inherently increases susceptibility to unmeasured confounding and selection bias compared with RCTs. Consequently, our pooled estimates may be influenced by heterogeneous reporting standards across real-world centers. In addition, our rigorous chronologic search cutoff (January 7, 2026) precluded the quantitative inclusion of the recently published ADVANTAGE AF trial. Furthermore, the absence of head-to-head comparative data with thermal ablation within our pooled cohorts precludes any definitive claims of superiority. Second, although the overall pooled cohort was robust, certain subgroup analyses were constrained by small sample sizes. Specifically, our key finding regarding the protective effect of prophylactic NTG injection is based on only 3 nonrandomized observational studies comprising 293 patients. Consequently, this subgroup analysis is inherently exploratory and hypothesis-generating, and these results should be interpreted with caution until validated by larger prospective randomized trials. Third, significant methodologic heterogeneity existed across centers regarding institutional PFA protocols, lesion validation end points, and NTG administration regimens (including timing and cumulative dosage). Fourth, given that PFA is an emerging technology, variable operator proficiency and the inherent learning curve across studies may have confounded procedural metrics (eg, ablation times) and periprocedural complication rates. Finally, although short- and medium-term outcomes are promising, the long-term durability of PFA for CTI ablation remains to be definitively established. Future large-scale, multicenter RCTs with extended follow-up are required to corroborate the efficacy and safety profiles observed in this analysis.

## Conclusion

This meta-analysis shows that PFA is an effective, rapid, and safe treatment for cavotricuspid isthmus-dependent atrial flutter. Although the electric field characteristics of PFA pose a risk for transient RCA spasm, our data suggest this complication may be mitigated with prophylactic administration of high-dose (>1 mg) intravenous NTG across different catheter platforms. These hypothesis-generating findings indicate that high-dose NTG represents a promising prophylactic strategy, although its establishment as a standardized frontline protocol requires further validation in large-scale randomized trials. Due to its unparalleled safety profile, PFA holds great clinical promise. However, large-scale randomized trials are needed to confirm its long-term durability.

## Protocol

The study protocol was prospectively registered in National Institute for Health and Care Research (https://www.crd.york.ac.uk/).

## Disclosures

The authors have no conflicts of interest to disclose.

## Footnotes

Appendix Supplementary data associated with this article can be found in the online version at https://doi.org/10.1016/j.hroo.2026.04.030
