# Supplementary material for: Efficacy and safety of pulsed field ablation for cavotricuspid isthmus-dependent atrial flutter: A meta-analysis based on real-world evidence
Source: Heart Rhythm O2. 2026 May 13;7(8):1428–39. doi: 10.1016/j.hroo.2026.04.030 (PMC13523579; doi:10.1016/j.hroo.2026.04.030)
Supplement: Supplementary file 1 [file mmc1.docx]

**Supplementary Table 1. Leave-one-out sensitivity analysis**

| **Omitted study** | **Pooled incidence** | **95% CI** | **I² (%)** |
| --- | --- | --- | --- |
| Farnir et al. 2025 | 0.018 | 0.008–0.029 | 78.7 |
| Demian et al. 2025 | 0.021 | 0.009–0.032 | 80.2 |
| Chaumont et al. 2024 | 0.020 | 0.010–0.030 | 80.6 |
| Juan et al. 2024 | 0.020 | 0.009–0.032 | 80.8 |
| Tam et al. 2025 | 0.020 | 0.010–0.030 | 80.7 |
| Reddy et al. 2022 | 0.013 | 0.007–0.018 | 34.4 |
| Andria et al. 2026 | 0.019 | 0.009–0.029 | 80.9 |
| Reddy et al. 2025 | 0.021 | 0.011–0.032 | 76.5 |
| Ruwald et al. 2024 | 0.018 | 0.008–0.027 | 79.6 |

Note: Sequential exclusion of individual studies did not materially alter the pooled incidence estimates, indicating robustness of the primary analysis.

**Figure S1. PRISMA flow diagram of the study selection process.**

The flowchart details the literature search, screening, and selection steps for the systematic review and meta-analysis evaluating the efficacy and safety of PFA for tricuspid isthmus-dependent atrial flutter.

Abbreviations: PRISMA, Preferred Reporting Items for Systematic Reviews and Meta-Analyses.

**Figure S2. The risk of bias assessments of the included studies.**

The "traffic light" plot details the specific quality judgments across the 10 domains of the Joanna Briggs Institute (JBI) critical appraisal checklist for each individual study. Teal indicates a low risk of bias, while yellow indicates some concerns.

Abbreviations: JBI, Joanna Briggs Institute.

**Supplementary Figure S3. Sensitivity analyses of subgroup findings for intraprocedural coronary artery spasm.**
(A) Forest plot presenting the sensitivity analysis stratified by prophylactic nitroglycerin dosage. The high-dose strategy (>1 mg) consistently demonstrated a significantly lower incidence of coronary artery spasm compared with the low-dose strategy (≤1 mg) (p = 0.0095).
(B) Forest plot illustrating the sensitivity analysis by ablation catheter type, conducted after excluding the primary source of heterogeneity (Reddy et al., 2022) and incorporating the Varipulse catheter as an additional category. Statistical heterogeneity was fully resolved (I² = 0%), and no significant differences in spasm incidence were observed among focal, pentaspline, and Varipulse catheter groups (p = 0.8807).

**Supplementary Figure S4. Pooled incidence of other major periprocedural complications associated with PFA.**
Forest plots illustrating the pooled incidence of (A) atrioventricular (AV) block, (B) pericardial effusion or tamponade, and (C) cardiovascular mortality across the included studies.

(A) The pooled incidence of AV block was low (0.00; 95% CI: 0.00–0.01).
(B, C) No cases of pericardial effusion/tamponade or cardiovascular mortality were reported, yielding a pooled incidence of 0.00 (95% CI: 0.00–0.00).

Arrows indicate 95% confidence intervals extending beyond the visual axis limit of 0.10.
Given the rarity of events, including studies with zero events, the Freeman–Tukey double arcsine transformation was applied to stabilize variances and calculate 95% confidence intervals.

doc
